# Robotic Surgical Management of Complicated Diverticulitis

**DOI:** 10.1007/s40719-025-00289-z

**Published:** 2025-06-27

**Authors:** Prisca C. Obidike, William J. Lain, Sook C. Hoang

**Affiliations:** 1 Department of Surgery, University of Virginia, 1215 Lee Street, Charlottesville, VA 22903 USA; 2 University of Virginia School of Medicine, Charlottesville, VA USA

**Keywords:** Diverticulitis, Diverticular disease, Complicated diverticulitis, Minimally invasive surgery, Robotic surgery

## Abstract

**Purpose of Review:**

Complicated diverticulitis is a common gastrointestinal pathology. Historically, surgical management involved multi-stage open resection and stoma creation, which were often associated with more significant morbidity, low stoma reversal rates, and postoperative complications.

**Recent Findings:**

Minimally invasive techniques, including laparoscopy and robotics, have emerged as alternative options for surgically managing complicated diverticulitis. While high conversion rates may deter surgeons from minimally invasive approaches, robotics offer several technical advantages, including three-dimensional visualization, increased instrument range of motion, reduced conversion rates compared to laparoscopy, and improved postoperative patient outcomes.

**Summary:**

In this review, we discuss robotic surgery as a safe and feasible approach to the surgical management of complicated diverticulitis both electively and emergently in select patients. We present recommendations for intraoperative robotics setup and patient positioning and propose solutions that address the limitations of robotics, such as longer operative times and specialized training, that affect the adoption of robotics for surgically managing complicated diverticulitis.

## Introduction

 Colonic diverticular disease is highly prevalent in industrialized nations and remains a significant clinical and economic burden. In the United States (US), there are over 200,000 hospitalizations and 300,000 emergency department visits for diverticular disease each year, resulting in over $2 billion in healthcare spending [ 1, 2 ]. Diverticular disease ranges from asymptomatic diverticulosis, defined as the presence of uninflamed outpouchings of the colonic wall, to uncomplicated and complicated diverticulitis [ 3 ]. Complicated diverticulitis is defined by abscess formation, free perforation associated with purulent or feculent peritonitis, stricture, and fistula formation [ 3, 4 ]. While uncomplicated diverticulitis can be managed medically with analgesics, antibiotics, and lifestyle changes, managing complicated diverticulitis may require hospitalization and surgical intervention [ 3, 5 ].

The decision to perform elective surgery for uncomplicated and complicated diverticulitis is a complex decision that requires shared decision-making between the patient and provider. Patient age and number of uncomplicated diverticulitis attacks are no longer strict criteria for surgical intervention. And while early surgical intervention was previously recommended to avoid recurrent episodes of complicated diverticulitis, recent modifications to the American Society of Colon and Rectal Surgeons (ASCRS) guidelines suggest an individualized approach for operative indication [6, 7]. The choice and mode of surgery for managing acute diverticulitis are also changing. Traditionally, surgical management of complicated diverticulitis involved open resections to allow visualization of the operative field. However, there is a growing adoption of minimally invasive techniques, including laparoscopic and robotic approaches, in the surgical management of complicated diverticulitis. Additionally, Hartmann’s procedure, the standard of care management for acute diverticulitis, has now transitioned to more minimally invasive approaches such as laparoscopic lavage or minimally invasive resections. If a resection is performed, surgeons are favoring upfront anastomoses in select patients to minimize the morbidity of the subsequent surgeries. More recently, robotic surgery offers significant operative advantages in surgically managing acute diverticulitis. It allows minimal abdominal wall morbidity through smaller incisions, three-dimensional (3D) operative field visualization, increased instrument rotational range, and lower conversion rates than laparoscopy [8–10].

In this review, we discuss the role of the robotics platform in the surgical management of complicated diverticulitis. Although diverticulitis can affect all parts of the colon, we will focus on left-sided complicated diverticulitis. Specifically, we examine the indications for robotic surgery, its safety, feasibility, and drawbacks in the management of complicated diverticulitis in elective and emergent settings.

### Diagnosis of Acute Diverticulitis

Computed tomography (CT), preferably with oral or rectal contrast and intravenous (IV) contrast, is the diagnostic imaging of choice recommended by the American Gastroenterological Association [11, 12]. Signs of diverticulitis on CT include bowel wall thickening, pericolic fat stranding, and pericolic fluid collections, which may be associated with complications, including free wall perforation, contrast extravasation, and fistula. Magnetic resonance imaging (MRI) can be helpful in patients with contraindications to CT and in the diagnosis of colo-vesicular, vaginal, or cutaneous fistulas. CT remains the preferred diagnostic modality for acute diverticulitis due to its high sensitivity and specificity, which is reported to be greater than 97%, and its cost-effectiveness [13, 14].

The Hinchey and modified Hinchey classifications are the most commonly used tools for stratifying the severity of acute diverticulitis (Table 1). The modified Hinchey classification expands on the original by including mild, uncomplicated disease (stage 0) and differentiating between confined pericolic inflammation (stage Ia) and abscess (stage Ib) [15]. Due to the objective difficulty distinguishing between stages I and II in the modified Hinchey classification, the World Society of Emergency Surgery (WSES) and the American Association for the Surgery of Trauma (AAST) have recently proposed two classifications that focus on CT findings (Table 2) [16]. In a retrospective study of 597 patients from four medical centers between 2014 and 2021, Cremonini et al. found the three classification systems (modified Hinchey, WSES, and AAST) were similarly effective in predicting complications, mortality rate, and the need for surgical intervention and reintervention [16].

**Table 1:** Hinchey and Modified Hinchey classifications

Hinchey	Modified Hinchey
	**Stage 0**– Mild, uncomplicated acute diverticulitis
**Stage I**– Localized abscess (pericolic or mesenteric abscess/phlegmon)	**Stage Ia**– Confined pericolic inflammation (phlegmon)
	**Stage Ib**– Confined pericolic abscess
**Stage II**– Pelvic intra-abdominal or retroperitoneal abscess	**Stage II**– Pelvic, distant intra-abdominal, or intraperitoneal abscess
**Stage III**– Purulent peritonitis	**Stage III**– Generalized purulent peritonitis
**Stage IV**– Feculent peritonitis	**Stage IV**– Feculent peritonitis

**Table 2:** World society of emergency surgery and the american association for the surgery of trauma classifications

WSES	AAST
**Stage 0**– Diverticula, thickening of the colonic wall, increased density of the pericolic fat	**Grade I**– Colonic inflammation
**Stage Ia**– Small pericolic air bubbles or some pericolic fluid without abscess	**Grade II**– Colon micro-perforation or pericolic phlegmon without abscess
**Stage Ib**– Abscesses ≤ 4 cm (without distant free gas)	**Grade III**– Localized pericolic abscess
**Stage IIa**– Abscess > 4 cm (without distant free gas)	**Grade IV**– Distant and/or multiple abscesses
**Stage IIb**– Distant gas (> 5 cm from inflamed bowel segment)	
**Stage III**– Diffuse fluid without distant free, diffuse gas	**Grade V**– Free colonic perforation with generalized peritonitis
**Stage IV**– Diffuse fluid with distant free, diffuse gas	

### Nonoperative Management of Diverticulitis

Uncomplicated acute diverticulitis can be treated with bowel rest, analgesics, antibiotics, and lifestyle changes. The ASCRS recommends hospitalization for patients with poor tolerance of oral intake, peritonitis on abdominal exam, immunosuppression, or advanced age [7]. Complicated diverticulitis is evidence of diverticulitis associated with the formation of abscess, fistula, bowel obstruction, or free perforation [17]. The treatment of complicated diverticulitis is complex and depends on the patient’s clinical and physical findings, comorbidities, surgical history, and the severity of the disease based on radiographic classification systems. Surgical intervention is indicated in patients with complicated diverticulitis with peritonitis or hemodynamic instability.

Abscess formation occurs in 15–40% of patients with complicated diverticulitis. Nonoperative treatment using antibiotics alone or with percutaneous drainage can successfully treat up to 80% of these cases. Current ASCRS guidelines recommend image-guided percutaneous drainage for hemodynamically stable patients with > 3 cm-sized abscesses. Patients who cannot undergo percutaneous drainage or fail to respond to medical treatment should be considered for surgery, which may include laparoscopic abscess drainage or surgical resection [7, 18, 19]. After the resolution of an episode of acute complicated diverticulitis, a follow-up colonoscopy is advised due to the elevated risk of occult malignancy associated with complicated diverticulitis [20].

### Surgical Management of Complicated Diverticulitis

Patients with complicated diverticulitis managed nonoperatively are at an increased risk of recurrent episodes [12]. While the 2014 ASCRS guidelines previously recommended interval elective colectomy after recovery from one episode of complicated diverticulitis to avoid potential recurrences, more recent studies have demonstrated successful medical treatment of recurrences. Updated ASCRS guidelines recommend that surgical intervention be individualized and weighed against the patient’s risk of surgery [7]. Additionally, recent studies on patients’ health-related quality of life may be useful to consider during the shared decision-making process regarding the decision to perform interval colectomy after successful nonoperative management. The LASER (Laparoscopic Elective Sigmoid Resection Following Diverticulitis) multi-center randomized controlled trial comparing laparoscopic elective sigmoid resection to conservative treatment in patients with recurrent, complicated, or persistent disease. They found a significantly higher increase in the Gastrointestinal Quality of Life Index in those patients who underwent sigmoid resection vs. successful conservative treatment (11.8 points vs. 0.2) [21]. Elective surgical treatment for complicated diverticulitis includes resection of the sigmoid colon followed by a colorectal anastomosis [7]. In patients with Hinchey III and IV diverticulitis, resection and colostomy (Hartmann’s procedure, HP) or primary resection and anastomosis (PRA) with or without a diverting stoma are possible surgical options [22]. Alternatively, laparoscopic lavage has been extensively studied for managing patients with Hinchey III diverticulitis who present in the acute setting. There are three major randomized control trials comparing resection to laparoscopic lavage for Hinchey III diverticulitis [23–25]. Although the outcomes measured varied across these trials, there was an overall increased risk of unresolved infection and abscess formation. Surgeons who perform laparoscopic lavage should be prepared to offer a secondary intervention if needed for patients with unresolved infections [7]. Laparoscopic lavage should not be offered for patients with Hinchey IV diverticulitis, as this can increase the risk for reoperation [26].

HP, first involving primary resection and creation of the colostomy and colostomy reversal at a later stage, was traditionally hailed as the gold standard surgical management for Hinchey III and IV diverticulitis. However, the significant complication rates, such as wound infection and anastomotic dehiscence after colostomy reversal, have brought into question whether it remains the best surgical option [22, 27]. In addition, several studies have reported that 20–50% of patients undergoing HP never get their stomas reversed [22, 28, 29]. Some may argue that the significant complication rates with HP are a result of this option being performed in patients with hemodynamic instability and peritonitis [27]. However, as a result of the increased morbidity associated with both the index HP surgery and the colostomy reversal surgery, there is a growing shift towards PRA with or without diversion in hemodynamically more stable patients with Hinchey III and IV diverticulitis [30–32]. Patients who undergo PRA with or without a diverting stoma are found to have no difference in the morbidity associated with the index surgery compared to HP. There is, however, a significant reduction in morbidity associated with the stoma reversal surgery compared to HP, and significantly greater stoma-free rates at 12 months after the index surgery. Recent data demonstrate that PRA is a safe and feasible option in the acute setting and could be considered in immunocompetent and hemodynamically stable patients [33, 34].

Minimally invasive techniques, including laparoscopic and robotic surgery, have increasingly been adopted for the surgical management of complicated diverticulitis. Several studies comparing minimally invasive techniques to open have demonstrated reduced hospital length-of-stay, morbidity, and pain associated with minimally invasive approaches [27]. Turley et al. found fewer complications (26% vs. 41.7%) and shorter hospitalization (8.9 vs. 11.6 days) with laparoscopic HP but no significant survival benefit [35]. Cassini et al. reported lower morbidity and faster recovery with laparoscopic HP, with 92% of patients successfully undergoing reversal [36]. A systematic review by Cirocchi et al. found minor benefits in postoperative outcomes but noted potential selection bias; patients undergoing open surgery were often sicker [37]. Similarly, the Sigma Trial, a multicenter RCT of 104 patients undergoing elective surgery for symptomatic sigmoid diverticulitis, compared patients who underwent laparoscopic PRA. Laparoscopic sigmoid resection significantly reduced major complication rates (15.4% lower than open surgery), pain, and hospital stay, and improved quality of life, though it required longer operating times [38]. As minimally invasive techniques continue to evolve, the emergence of robotic-assisted surgery offers the potential to enhance precision further, reduce complications, and expand the benefits of minimally invasive approaches to a broader range of patients with diverticulitis.

### History of Robotics in Abdominal Surgery

Camera holders served as the first robots in surgical practice. In 1994, the U.S. Food and Drug Administration (FDA) approved the Automated Endoscope System for *O*ptimal Positioning (AESOP) as a robotic camera holder during surgery [39]. The AESOP robot maintained a stable camera platform for the surgeon performing laparoscopic procedures at the bedside. At its inception, the robot arm was manually or remotely controlled with a foot pedal or hand control, but was developed into a voice-controlled robot in later generations [39–41]. AESOP used as a camera holder was reported to have comparable operative times as laparoscopic operations, significantly steadier camera platforms than a human camera holder, and allowed for solo-surgeon laparoscopic operations [39, 42].

Over time, surgical robots evolved into telerobotic platforms that allowed surgeons to operate on patients from remote locations. In telerobotic operations, the surgeon sits at a computer console located remotely beside the patient, during which the computer translates the movement of the surgeon’s hands into corresponding motions of the robotic instruments [39, 41]. The telerobot platform offered numerous advantages over traditional laparoscopic techniques, including a 3D operative field view, a stable camera platform, increased instrumental motion range, and improved surgeon ergonomics. However, its early application in abdominal surgery was fraught with steep learning curves, bulky surgical arms and equipment, limited surgical instruments, and longer operative times [39, 42]. It is only recently, with significant technological advancements, that robotics surgery has had greater applications in abdominal surgeries.

### Robotic Approach for Diverticulitis in Elective and Emergent Settings

Robotic Surgery has expanded to become a feasible and comparable alternative to laparoscopy for complicated diverticulitis. The most commonly used da Vinci robot system offers technical advantages, including superior visualization, increased instrument range of motion, and ureteral identification with indocyanine green (ICG), allowing for better dissection around inflamed tissue [43]. These advantages likely contribute to the reduced conversion to open and comparable complication rates with robotics compared to laparoscopy. More recently, a systematic review and meta-analysis of fifteen studies involving 3711 robotic diverticular resections reported a lower conversion rate and no significant difference in major complications with robotic resections compared to laparoscopic resections [9]. A propensity score-matched analysis by Raskin et al. reported significantly fewer postoperative complications of ileus, wound complications, and acute renal failure in robotic sigmoidectomies than in laparoscopic and open [44]. Furthermore, the robotic approach was associated with significantly shorter hospital length-of-stay than the laparoscopic and open groups.

The use of robotic surgery for diverticulitis in the emergent setting is highly debated. Recent studies have demonstrated that robotics is safe, feasible, and comparable to laparoscopy for diverticulitis surgery, even in the emergent setting. Curfman et al. recently conducted a retrospective study of 2500 emergent colorectal surgeries for diverticulitis, comparing outcomes across 126 robotics, 446 laparoscopic, and 1952 open approaches [45]. Robotic approach was associated with a significant decrease in ICU admission, anastomotic leak rates, and borderline improvement in length of stay rates compared to open approaches. Compared to laparoscopy, robotics had comparable rates of ICU admission, ICU length of stay, readmission, mortality, and surgical site infection rates. Furthermore, robotics was associated with a significantly reduced conversion rate than laparoscopy (7.9% vs. 28.7%). Although surgeon experience could not be directly assessed, the average number of robotic surgeries performed per year was examined and found to vary significantly across groups. Surgeons who performed open emergent diverticular surgery averaged 16.2 robotic surgeries per year compared to 27.3 in the laparoscopic group and 63 in the robotics group. Therefore, the authors concluded that although robotics could be a safe and feasible alternative to laparoscopy, the improved outcomes in the robotics group may be secondary to increased surgeon comfort and experience.

Recently, Arnott et al. compared the short-term outcomes for 6583 non-elective laparoscopic and 297 robotic colectomies for diverticulitis between 2012 and 2019 in the American College of Surgeons-National Surgical Quality Improvement Program database [43]. They found no significant differences in mortality, anastomotic leak, surgical site infection, reoperation, readmission, or length of stay between treatment groups. Conversion rate was significantly lower in robotics (11.5% vs. 28.3%) than in the laparoscopic group. While there were similar comorbidities between both groups, the laparoscopic group had greater percentages of preoperative sepsis (31.6% vs. 10.8%) and emergency case status (32.3% vs. 6.7%). A 2022 consensus statement by WSES discussed the importance of careful patient selection, the availability of robot platforms after hours, and well-experienced surgeons and supporting staff, which are critical for the success of robotics utilization in emergent settings [46]. Although there could be an initial reluctance to implement robotic surgery for diverticulitis in the emergent setting, the decision to use robotics should be individualized and based on the patient’s status, surgeon comfort and experience, and institutional staff and robotics support.

### Technical Considerations and Patient Set-Up

As in all surgical procedures, regardless of approach, proper patient setup and positioning are key to a successful operation. For robotic sigmoid resections, the patient is placed on the surgical stretcher in a lithotomy position. We prefer using the pink pad device with additional straps to secure the chest. Both arms are tucked with all pressure points supported. Before docking the robot, the patient is positioned in steep Trendelenburg with the left side tilted up. This allows the small bowel to be reflected out of the pelvis and away from the sigmoid mesentery. If tolerated by the patient, we prefer 24 degrees Trendelenburg and 12 degrees tilt towards the patient’s right. The ‘tilt test’ is performed before prepping and draping the patient to ensure safe positioning. Once all OR staff, including the Anesthesiology team, are satisfied with the safety of positioning, the patient can then be prepped and draped sterilely. The authors prefer to enter the abdomen using a Veress needle technique. The first port is placed just to the right inferolateral of the umbilicus. An 8 mm robotic trocar is placed here for the camera. The daVinci Xi platform is set to the *lower abdominal* or *pelvis* configuration. The boom enters from the patient’s left, and the ports are placed diagonally from the right ASIS to the left costal margin. A 12 mm port is placed for arm four at the right lower-most port, which can accommodate a robotic stapler. The camera is placed at arm 3. The remaining arms 1,2, and 3 are 8 mm robotic trocars. An additional assist port is placed at the right upper abdomen, triangulating arms 3 and 4. If an air-seal device is used, the air-seal trocar is placed at the assist port site. If the surgeon anticipates the need to mobilize proximally toward the splenic flexure, arms 1 and 2 can be placed more diagonally to allow proximal mobilization of the descending colon and splenic flexure.

Once the robot is docked, the arms are targeted toward the sigmoid fossa. We prefer the following instruments:

Arm 1: tip up.

Arm 2: fenestrated bipolar.

Arm 3: robotic camera.

Arm 4: hook/cautery scissor/vessel sealer/stapler.

For the entirety of the case, the tip-up in arm 1 is used for retraction while arms 2 and 4 are the active operating arms. Once the specimen is resected, an intracorporeal or extracorporeal placement of the anvil can be performed. If an extracorporeal placement is performed, it can be done through the extraction site. A left lower quadrant or Pfannensteil extraction site is created, and a small wound protector is placed. Once the specimen is extracted and the anvil secured, the end-to-end circular stapled anastomosis is performed. Pneumoperitoneum is achieved by closure of the extraction site. Alternatively, we find that a 6-Ortho glove can fit easily around the standard wound protector and provides adequate tension to achieve pneumoperitoneum. Indocyanine green and the camera set to the firefly mode can be utilized to ensure adequate perfusion to the colon and rectum. Finally, a flexible sigmoidoscopy is performed to ensure that the anastomosis is air-tight.

### Limitations of Robotic Surgery for Complicated Diverticulitis

Increased cost, steep learning curve, and longer operating times are often cited as disadvantages of robotic surgeries compared to the laparoscopic approach. The implementation of a robotic surgical platform involves various costs, including acquisition and maintenance expenses, as well as costs associated with operating room personnel, operating room time, online and simulation training modules, and surgeon instrument preferences [47]. Although there are economic benefits from reduced patient length of stay, fewer complications, and lower readmission rates associated with robotic surgery, there is a lack of comprehensive data comparing the overall economic costs of robotics in colorectal surgery. A propensity score-matched analysis comparing the cost of open, laparoscopic, and robotic surgical treatment for colon cancer showed robotic surgery had a higher median cost than laparoscopic surgery [48]. The cost per procedure can be lowered by using robotics in high-volume multidisciplinary centers, advanced instruments with longer lifespans, and the economic benefits of reduced morbidity in robotic surgery patients [47, 49].

The success of robotic surgery for emergent cases relies on multiple factors. Thomas et al. concluded that a dedicated and well-trained surgical team and standardized protocols for robotic platform setup and docking are needed to implement robotic surgery in emergent settings [50]. In an ideal setting, all team members should have completed the robotic training modules and have adequate experience with robotic technology. However, this takes considerable time and effort from all team members, which may be a deterrent. Additionally, institutional support for the accessibility of the robotic platforms and a well-trained surgical team at night and on weekends is critical for the success of robotics in emergent settings.

Robotic surgeries have been reported to have longer operating times in both elective and emergent settings. Larkins et al. reported that robotic resections for diverticulitis had a significantly longer operative time on average, 185.9 min, than laparoscopic cases, 177.4 min [9]. Curfman et al. reported robotic resections for diverticulitis in the emergent settings were higher than laparoscopy and open approaches (262 vs. 207 vs. 182 min, respectively). Similarly, Raskin et al. showed significantly longer operating time with robotics than with laparoscopic and open approaches for elective sigmoidectomies [44]. They concluded that longer operating time could result from the steep learning curve with robotics, patient positioning, robot setup, and docking, and the quality of the assisting surgical team. Longer operative time of the robotic approach may present a challenge for its use in the emergent setting where rapid surgical intervention is critical. Efforts aimed at identifying and addressing areas that contribute to the longer operating time could be implemented through simulation training and the utilization of a dedicated robotic surgical team [51].

These logistical and institutional demands not only affect efficiency and implementation, but also underscore broader issues of access and equity in robotic surgery. Current literature highlights several disparities across patient populations. For instance, insurance status significantly impacts access. Patients with private insurance are more likely to receive robotic surgery than those with Medicaid or no insurance [52, 53]. Additionally, racial disparities persist. Specifically, Black and Hispanic patients are less likely to undergo robotic procedures compared to White patients [53–55]. Akram et al. found that Black patients were more likely to undergo open surgery than minimally invasive approaches for diverticular disease specifically [56]. Geographic location and hospital type also play key roles in access. Patients treated at academic or high-volume centers have significantly greater access to robotic surgery than those at community hospitals [53, 57]. Lemini et al. identified reduced odds of receiving MIS among older, sicker, rural, and Medicaid patients [58]. Moreover, robotic technology at a facility often determines access more strongly than individual socioeconomic status [59]. For example, in a study conducted by Armstrong et al., 23 surgeons with varied robotic experiences and from different practice settings identified access and availability as the most important systemic issues to address (followed by institutional support, cost, quality of supporting evidence, and need for robotic training) [60]. These patterns highlight the systemic nature of the barriers to advanced surgical care. Without deliberate efforts to address these disparities, the benefits of robotic surgery, including fewer complications, faster recovery, and improved outcomes, may remain limited to select populations. Policy interventions should aim to increase the availability of robotic platforms in diverse healthcare settings, ensure institutional support for surgical teams, and promote equitable reimbursement and training.

## Discussion

Diverticulitis treatment has shifted largely into medical management [17]. However, surgical intervention is still warranted in cases of complicated or recurrent disease. Historically, open surgery was the primary approach, especially in cases involving perforation, abscess formation, or bowel obstruction. Although these procedures are highly effective for managing complicated diverticulitis, they are associated with significant morbidity, such as longer recovery times, increased postoperative pain, and higher rates of complications like infection or bowel leakage [6]. Over the past two decades, minimally invasive techniques, particularly laparoscopic surgery, have become the preferred approach for managing both complicated and elective cases of diverticulitis. This has been shown to reduce postoperative complications, including infection, bleeding, and anastomotic leaks, as well as shortening hospital stays and recovery times. However, the success of laparoscopy in complicated cases, such as large abscesses or perforations, can depend on the surgeon’s experience and the extent of the disease. In such cases, these procedures may require conversion to open surgery if the situation becomes too complex to manage via minimally invasive techniques [22].

With the emergence of robotic surgery, particularly the da Vinci Surgical System, minimally invasive techniques have been further refined, with enhanced control, flexibility, and visualization during procedures, especially in challenging cases with dense adhesions or complex anatomy [61]. Furthermore, robotic systems can reduce the need for conversion to open surgery, which is particularly beneficial in patients with challenging diverticulitis. However, robotic surgery has its drawbacks, including its high cost, longer setup times, and the steep learning curve required for surgeons to master the robotic system. For less complicated cases, studies suggest that robotic surgery may not offer significant advantages over laparoscopic approaches [62].

## Conclusion

The management of complicated diverticulitis has evolved significantly over the past decade, with a shift towards more individualized approaches in surgical decision-making. While nonoperative management remains a viable option for many patients, the decision to pursue interval elective colectomy must be tailored to each patient’s clinical situation, considering factors such as severity of disease, recurrent episodes, comorbidities, and the patient’s overall quality of life. Minimally invasive techniques, including laparoscopic and robotic surgery, have shown promising benefits in terms of reduced complications, faster recovery, and improved outcomes compared to traditional open surgery. However, they come with challenges such as longer operating times and increased costs. The rise of robotic-assisted surgery presents new opportunities for enhancing surgical precision, but it also highlights disparities in access based on factors like insurance status, race, and geographical location. As surgical technologies continue to advance, addressing these systemic barriers will ensure that all patients can benefit from the latest surgical innovations. Future research and policy interventions should focus on improving access to minimally invasive procedures, optimizing surgical techniques, and promoting equitable care across diverse patient populations.

## Data Availability

No datasets were generated or analysed during the current study.

## Key References

1. Curfman, K.R., Jones, I.F., Conner, J.R. et al. Robotic colorectal surgery in the emergent diverticulitis setting: is it safe? A review of large national database. Int J Colorectal Dis 38, 142 (2023). 10.1007/s00384-023-04436-3.
  - An extensive retrospective review evaluated the safety and outcomes of using robotic surgery for emergent diverticulitis cases compared to laparoscopic and open approaches. The robotic approach was associated with significantly reduced ICU admission, anastomotic leak rates, and overall length of stay.
2. Panin, S.I., Nechay, T.V., Sazhin, A.V. et al. Should we encourage the use of robotic technologies in complicated diverticulitis? Results of systemic review and meta-analysis. Front. Robot. Ai, 10(2023). 10.3389/frobt.2023.1208611.
  - This systematic review and meta-analysis compared robotic and laparoscopic surgery for complicated diverticulitis from five non-randomized studies. This study suggests that robotic surgery is an appropriate option for managing complicated diverticulitis. However, high-quality randomized trials are needed to support widespread adoption of robotics for complicated diverticulitis.
3. Ali F, Raskin E. Robotic Surgery for Complicated Diverticular Disease. Clin Colon Rectal Surg. 2021 Sep;34 [5]:297–301. doi: 10.1055/s-0041-1729863.
  - This narrative review discusses the advantages of robotic surgery and its usefulness in managing anatomical challenges posed by complicated diverticulitis. It examines technical strategies for managing complex diverticular cases, including colo-vesicular, vaginal, and cutaneous fistulae.

## References

[1] Strate LL, Morris AM. Epidemiology, pathophysiology, and treatment of diverticulitis. Gastroenterology. 2019;156(5):1282–e12981. 30660732 10.1053/j.gastro.2018.12.033 PMC6716971

[2] Imaeda H, Hibi T. The burden of diverticular disease and its complications: West versus East. Inflamm Intest Dis. 2018;3(2):61–8. 30733949 10.1159/000492178 PMC6361582

[3] Talutis SD, Kuhnen FAH. Pathophysiology and epidemiology of diverticular disease. Clin Colon Rectal Surg. 2021;34(2):81–5. 33642946 10.1055/s-0040-1716698 PMC7904337

[4] Jacobs DO, Diverticulitis. N Engl J Med. 2007;357(20):2057–66. 18003962 10.1056/NEJMcp073228

[5] Daniels L, Philipszoon LE, Boermeester MA. A hypothesis: important role for gut microbiota in the etiopathogenesis of diverticular disease. Dis Colon Rectum. 2014;57(4):539–43. 24608313 10.1097/DCR.0000000000000078

[6] Hawkins AT, Wise PE, Chan T, Lee JT, Glyn T, Wood V, et al. Diverticulitis: an update from the age old paradigm. Curr Probl Surg. 2020;57(10):100862. 33077029 10.1016/j.cpsurg.2020.100862 PMC7575828

[7] Hall J, Hardiman K, Lee S, Lightner A, Stocchi L, Paquette IM, et al. The American society of Colon and rectal surgeons clinical practice guidelines for the treatment of Left-Sided colonic diverticulitis. Dis Colon Rectum. 2020;63(6):728–47. 32384404 10.1097/DCR.0000000000001679

[8] Williams E, Prabhakaran S, Kong JC, Bell S, Warrier SK, Simpson P, et al. Utility of intra-operative flexible sigmoidoscopy to assess colorectal anastomosis: a systematic review and meta-analysis. ANZ J Surg. 2021;91(4):546–52. 33021045 10.1111/ans.16338

[9] Larkins K, Mohan H, Apte SS, Chen V, Rajkomar A, Larach JT, et al. A systematic review and meta-analysis of robotic resections for diverticular disease. Colorectal Dis. 2022;24(10):1105–16. 35723895 10.1111/codi.16227

[10] Bromley L, Huang D, Mohan H, Rajkomar A, Larach JT, Heriot A, et al. Feasibility and safety of a robotic approach to diverticular disease: a retrospective series of short-term outcomes. ANZ J Surg. 2023;93(6):1626–30. 36629147 10.1111/ans.18259

[11] Bailey J. Diverticular Disease: Rapid Evid Rev. 2022;106(2). 35977135

[12] Peery AF, Shaukat A, Strate LL. AGA clinical practice update on medical management of colonic diverticulitis. Expert Rev Gastroenterol. 2021;160(3):906–e9111. 10.1053/j.gastro.2020.09.059 PMC7878331 33279517

[13] Linzay CD, Pandit S. Acute Diverticulitis. StatPearls Publishing [Internet]. 2023; Available from: https://www.ncbi.nlm.nih.gov/books/NBK459316/29083630

[14] Naves ADA, D’Ippolito G, Souza LRMF, Borges SP, Fernandes GM. What radiologists should know about tomographic evaluation of acute diverticulitis of the colon. Radiol Bras. 2017;50(2):126–31. 28428656 10.1590/0100-3984.2015.0227 PMC5397004

[15] Cremonini C, Biloslavo A, Robustelli V, Giannessi S, Rossi Del Monte S, Mastronardi M, et al. What are the differences between the three most used classifications for acute colonic diverticulitis? A comparative multicenter study. J Trauma Acute Care Surg. 2024;96(2):326–31. 37661307 10.1097/TA.0000000000004133 PMC11444344

[16] Cremonini C, Biloslavo A, Robustelli V, Giannessi S, Rossi Del Monte S, Mastronardi M, et al. What are the differences between the three most used classifications for acute colonic diverticulitis? A comparative multicenter study. J Trauma Acute Care Surg. 2024;96(2):326. 37661307 10.1097/TA.0000000000004133 PMC11444344

[17] Sacks OA, Hall J. Management of diverticulitis: A review. JAMA Surg. 2024;159(6):696. 38630452 10.1001/jamasurg.2023.8104

[18] Aquina CT, Becerra AZ, Xu Z, Justiniano CF, Noyes K, Monson JRT, et al. Population-based study of outcomes following an initial acute diverticular abscess. J Br Surg. 2019;106(4):467–76. 10.1002/bjs.10982 30335195

[19] Lambrichts DPV, Van Der Bolkenstein HE, Dieleman D, Crolla RMPH, Dekker JWT, et al. Multicentre study of non-surgical management of diverticulitis with abscess formation. Br J Surg. 2019;106(4):458–66. 30811050 10.1002/bjs.11129 PMC6593757

[20] Meyer J, Orci LA, Combescure C, Balaphas A, Morel P, Buchs NC, et al. Risk of colorectal Cancer in patients with acute diverticulitis: A systematic review and Meta-analysis of observational studies. Clin Gastroenterol Hepatol. 2019;17(8):1448–e145617. 30056181 10.1016/j.cgh.2018.07.031

[21] Santos A, Mentula P, Pinta T, Ismail S, Rautio T, Juusela R, et al. Comparing laparoscopic elective sigmoid resection with Conservative treatment in improving quality of life of patients with diverticulitis: the laparoscopic elective sigmoid resection following diverticulitis (LASER) randomized clinical trial. JAMA Surg. 2021;156(2):129. 33206182 10.1001/jamasurg.2020.5151 PMC7675217

[22] Constantinides VA, Heriot A, Remzi F, Darzi A, Senapati A, Fazio VW, et al. Operative strategies for diverticular peritonitis: a decision analysis between primary resection and anastomosis versus Hartmann’s procedures. Ann Surg. 2007;245(1):94–103. 17197971 10.1097/01.sla.0000225357.82218.ce PMC1867925

[23] Rodriguez GR, Reed RN, Brody F, Duncan JE. Less is (Sometimes) more: laparoscopic peritoneal lavage and drainage for diverticulitis. J Laparoendosc Adv Surg Tech. 2024;34(11):962–6. 10.1089/lap.2024.0328 39411778

[24] Kohl A, Rosenberg J, Bock D, Bisgaard T, Skullman S, Thornell A, et al. Two-year results of the randomized clinical trial DILALA comparing laparoscopic lavage with resection as treatment for perforated diverticulitis. Br J Surg. 2018;105(9):1128–34. 29663316 10.1002/bjs.10839 PMC6055876

[25] Azhar N, Johanssen A, Sundström T, Folkesson J, Wallon C, Kørner H, et al. Laparoscopic lavage vs primary resection for acute perforated diverticulitis: Long-term outcomes from the Scandinavian diverticulitis (SCANDIV) randomized clinical trial. JAMA Surg. 2021;156(2):121. 33355658 10.1001/jamasurg.2020.5618 PMC7758831

[26] Kiely MX, Yao M, Chen L. Laparoscopic lavage in the management of Hinchey III/IV diverticulitis. Clin Colon Rectal Surg. 2021;34(2):104–12. 33642950 10.1055/s-0040-1716702 PMC7904334

[27] Costantini R. Hartmann’s procedure for complicated diverticulitis: A critical reappraisal. TOATJ. 2019;13(1):121–31.

[28] Regenet N, Pessaux P, Hennekinne S, Lermite E, Tuech JJ, Brehant O, et al. Primary anastomosis after intraoperative colonic lavage vs. Hartmann’s procedure in generalized peritonitis complicating diverticular disease of the colon. Int J Colorectal Dis. 2003;18(6):503–7. 12910361 10.1007/s00384-003-0512-1

[29] Schilling MK, Maurer CA, Kollmar O, Büchler MW. Primary vs. secondary anastomosis after sigmoid colon resection for perforated diverticulitis (Hinchey stage III and IV): A prospective outcome and cost analysis. Dis Colon Rectum. 2001;44(5):699–703. 11357032 10.1007/BF02234569

[30] Oberkofler CE, Rickenbacher A, Raptis DA, Lehmann K, Villiger P, Buchli C, et al. A multicenter randomized clinical trial of primary anastomosis or Hartmann’s procedure for perforated left colonic diverticulitis with purulent or fecal peritonitis. Ann Surg. 2012;256(5):819–26. discussion 826–827. 23095627 10.1097/SLA.0b013e31827324ba

[31] Swank HA, Vermeulen J, Lange JF, van der Mulder IM, Stassen LP, et al. The ladies trial: laparoscopic peritoneal lavage or resection for purulent peritonitisa and Hartmann’s procedure or resection with primary anastomosis for purulent or faecal peritonitisb in perforated diverticulitis (NTR2037). BMC Surg. 2010;10:29. 20955571 10.1186/1471-2482-10-29 PMC2974662

[32] Bridoux V, Regimbeau JM, Ouaissi M, Mathonnet M, Mauvais F, Houivet E, et al. Hartmann’s procedure or primary anastomosis for generalized peritonitis due to perforated diverticulitis: A prospective multicenter randomized trial (DIVERTI). J Am Coll Surg. 2017;225(6):798–805. 28943323 10.1016/j.jamcollsurg.2017.09.004

[33] Lee JM, P Chang JB, Hechi ME, Kongkaewpaisan N, Bonde A, Mendoza AE, et al. Hartmann’s procedure vs primary anastomosis with diverting loop ileostomy for acute diverticulitis: nationwide analysis of 2,729 emergency surgery patients. J Am Coll Surg. 2019;229(1):48–55. 30902639 10.1016/j.jamcollsurg.2019.03.007

[34] Wu S, Al Khaldi M, Richard CS, Dagbert F, Diverticulitis. A review of current and emerging Practice-Changing evidence. Clin Colon Rectal Surg. 2024;37(6):359–67. 39399131 10.1055/s-0043-1777439 PMC11466519

[35] Turley RS, Barbas AS, Lidsky ME, Mantyh CR, Migaly J, Scarborough JE. Laparoscopic versus open Hartmann procedure for the emergency treatment of diverticulitis: a propensity-matched analysis. Dis Colon Rectum. 2013;56(1):72–82. 23222283 10.1097/DCR.0b013e3182749cf5 PMC4431891

[36] Cassini D, Miccini M, Manoochehri F, Gregori M, Baldazzi G. Emergency Hartmann’s procedure and its reversal: A totally laparoscopic 2-Step surgery for the treatment of Hinchey III and IV diverticulitis. Surg Innov. 2017;24(6):557–65. 28748737 10.1177/1553350617722226

[37] Cirocchi R, Fearnhead N, Vettoretto N, Cassini D, Popivanov G, Henry BM, et al. The role of emergency laparoscopic colectomy for complicated sigmoid diverticulits: A systematic review and meta-analysis. Surgeon. 2019;17(6):360–9. 30314956 10.1016/j.surge.2018.08.010

[38] Klarenbeek BR, Veenhof AA, Van Der Bergamaschi R, De Lange ES, et al. Laparoscopic sigmoid resection for diverticulitis decreases major morbidity rates: A randomized control trial: Short-term results of the Sigma trial. Ann Surg. 2009;249(1):39–44. 19106674 10.1097/SLA.0b013e31818e416a

[39] Ballantyne GH. Robotic surgery, telerobotic surgery, telepresence, and Telementoring. Review of early clinical results. Surg Endosc. 2002;16(10):1389–402. 12140630 10.1007/s00464-001-8283-7

[40] Sackier JM, Wang Y. Robotically assisted laparoscopic surgery. From concept to development. Surg Endosc. 1994;8(1):63–6. 8153867 10.1007/BF02909496

[41] Pugin F, Bucher P, Morel P. History of robotic surgery: from AESOP^®^ and ZEUS^®^ to Da Vinci^®^. J Visc Surg. 2011;148(5):e3–8. 21974854 10.1016/j.jviscsurg.2011.04.007

[42] Ballantyne GH. The pitfalls of laparoscopic surgery: challenges for robotics and telerobotic surgery: surgical laparoscopy. Endoscopy Percutaneous Techniques. 2002;12(1):1–5. 10.1097/00129689-200202000-00001 12008756

[43] Arnott SM, Arnautovic A, Haviland S, Ng M, Obias V. Safety of robotic surgical management of non-elective colectomies for diverticulitis compared to laparoscopic surgery. J Robotic Surg. 2022;17(2):587–95. 10.1007/s11701-022-01452-3 36048320

[44] Raskin ER, Keller DS, Gorrepati ML, Akiel-fu S, Mehendale S, Cleary RK. Propensity-Matched analysis of sigmoidectomies for diverticular disease. JSLS. 2019;23(1):e201800073. 10.4293/JSLS.2018.00073 PMC6328361 30675092

[45] Curfman KR, Jones IF, Conner JR, Neighorn CC, Wilson RK, Rashidi L. Robotic colorectal surgery in the emergent diverticulitis setting: is it safe? A review of large National database. Int J Colorectal Dis. 2023;38(1):142. 37225935 10.1007/s00384-023-04436-3

[46] de’Angelis N, Khan J, Marchegiani F, Bianchi G, Aisoni F, Alberti D, et al. Robotic surgery in emergency setting: 2021 WSES position paper. World J Emerg Surg. 2022;17(1):4. 35057836 10.1186/s13017-022-00410-6 PMC8781145

[47] Marano A, Borghi F. Costs in Robotic Colorectal Surgery. In: Ceccarelli G, Coratti A, editors. Robotic Surgery of Colon and Rectum [Internet]. Cham: Springer International Publishing; 2024 [cited 2025 Mar 16]. pp. 25–31. (Updates in Surgery). Available from: https://link.springer.com/10.1007/978-3-031-33020-9_4

[48] Chiu CC, Hsu WT, Choi JJ, Galm B, Lee Mtse, Chang G. Comparison of outcome and cost between the open, laparoscopic, and robotic surgical treatments for colon cancer: a propensity score-matched analysis using nationwide hospital record database. Surg Endosc. 2019;33(11):3757–65. 30675661 10.1007/s00464-019-06672-7

[49] Patel S, Rovers MM, Sedelaar MJP, Zusterzeel PLM, Verhagen AFTM, Rosman C, et al. How can robot-assisted surgery provide value for money? BMJ Surg Interv Health Technol. 2021;3(1):e000042. 35047798 10.1136/bmjsit-2020-000042 PMC8647572

[50] Thomas A, Altaf K, Sochorova D, Gur U, Parvaiz A, Ahmed S. Effective implementation and adaptation of structured robotic colorectal programme in a busy tertiary unit. J Robotic Surg. 2021;15(5):731–9. 10.1007/s11701-020-01169-1 PMC8423644 33141410

[51] Lasser MS, Patel CK, Elsamra SE, Renzulli JF, Haleblian GE, Pareek G. Dedicated robotics team reduces pre-surgical Preparation time. Indian J Urol. 2012;28(3):263–6. 23204651 10.4103/0970-1591.102696 PMC3507392

[52] Childers CP, Uppal A, Tillman M, Chang GJ, Tran Cao HS. Insurance disparities in access to robotic surgery for colorectal Cancer. Ann Surg Oncol. 2023;30(6):3560–8. 36943527 10.1245/s10434-023-13354-1

[53] Hayden DM, Korous KM, Brooks E, Tuuhetaufa F, King-Mullins EM, Martin AM, et al. Factors contributing to the utilization of robotic colorectal surgery: a systematic review and meta-analysis. Surg Endosc. 2023;37(5):3306–20. 36520224 10.1007/s00464-022-09793-8 PMC10947550

[54] Kim SP, Boorjian SA, Shah ND, Weight CJ, Tilburt JC, Han LC, et al. Disparities in access to hospitals with robotic surgery for patients with prostate cancer undergoing radical prostatectomy. J Urol. 2013;189(2):514–20. 23253307 10.1016/j.juro.2012.09.033

[55] Logan CD, Mahenthiran AK, Siddiqui MR, French DD, Hudnall MT, Patel HD, et al. Disparities in access to robotic technology and perioperative outcomes among patients treated with radical prostatectomy. J Surg Oncol. 2023;128(2):375–84. 37036165 10.1002/jso.27274 PMC10330024

[56] Akram WM, Vohra N, Irish W, Zervos EE, Wong J. Racial disparity in the surgical management of diverticular disease. Am Surg. 2022;88(5):929–35. 34964694 10.1177/00031348211058623

[57] Tatarian T, McPartland C, Nie L, Yang J, Spaniolas K, Docimo S, et al. Socioeconomic disparities in the utilization of primary robotic hernia repair. Surg Endosc. 2023;37(6):4829–33. 36138250 10.1007/s00464-022-09627-7

[58] Lemini R, Spaulding AC, Osagiede O, Cochuyt JJ, Naessens JM, Crandall M, et al. Disparities in elective surgery for diverticulitis: identifying the gap in care. Am J Surg. 2019;218(5):899–906. 30878216 10.1016/j.amjsurg.2019.03.001

[59] Perkins LA, Santorelli JE, Black KM, Adams LM, Jacobsen G, Liepert AE, et al. Robotic availability, not payor status, determines access to robotic emergency general surgery hernia repair in California and Florida. Surg Endosc. 2024;38(11):6923–9. 39390233 10.1007/s00464-024-11283-y

[60] Armstrong M, Lu P, Wang J, El-Hayek K, Cleary S, Asbun H, et al. Advancing minimally invasive hepato-pancreato-biliary surgery: barriers to adoption and equitable access. Surg Endosc. 2024;38(10):5643–50. 39117957 10.1007/s00464-024-11078-1 PMC11980437

[61] Ali F, Raskin E. Robotic surgery for complicated diverticular disease. Clin Colon Rectal Surg. 2021;34(05):297–301. 34504402 10.1055/s-0041-1729863 PMC8416326

[62] Presl J, Ehgartner M, Schabl L, Singhartinger F, Gantschnigg A, Wallner E, et al. Robotic surgery versus conventional laparoscopy in sigmoid colectomy for diverticular disease-a comparison of operative trauma and cost-effectiveness: retrospective, single-center analysis. Langenbecks Arch Surg. 2024;409(1):200. 38935194 10.1007/s00423-024-03382-0 PMC11211106

